# Supplementary material for: Oxygen Concentration Plays a Critical Role in Fibrinogen-Mediated Platelet Activation via Inactivation of αIIbβ3 and Modulation of Fibrinogen
Source: Biomolecules. 2025 Mar 29;15(4):501. doi: 10.3390/biom15040501 (PMC12024578; doi:10.3390/biom15040501)
Supplement: Supplementary file 1 [file biomolecules-15-00501-s001.zip › biomolecules-3320306-supplementary.pdf]

Supplementary Figures

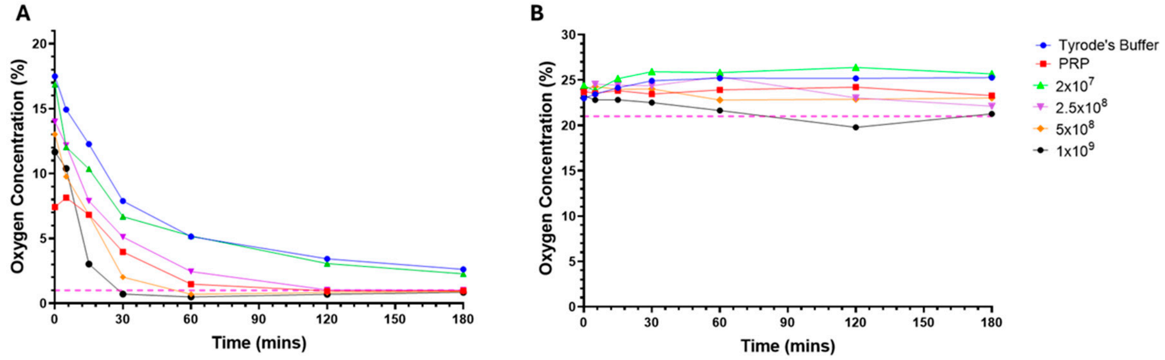

Figure S1: Establishing experimental parameters. *Oxygen concentration of washed platelets at a range of concentrations (2x10*^{7}*-1x10*^{9} *platelets/ml), PRP and Tyrode's buffer was monitored over 3 hours. (A) oxygen concentration over time in the hypoxia chamber set to 1%* O_2*, (B) oxygen concentration over time in normoxia.*

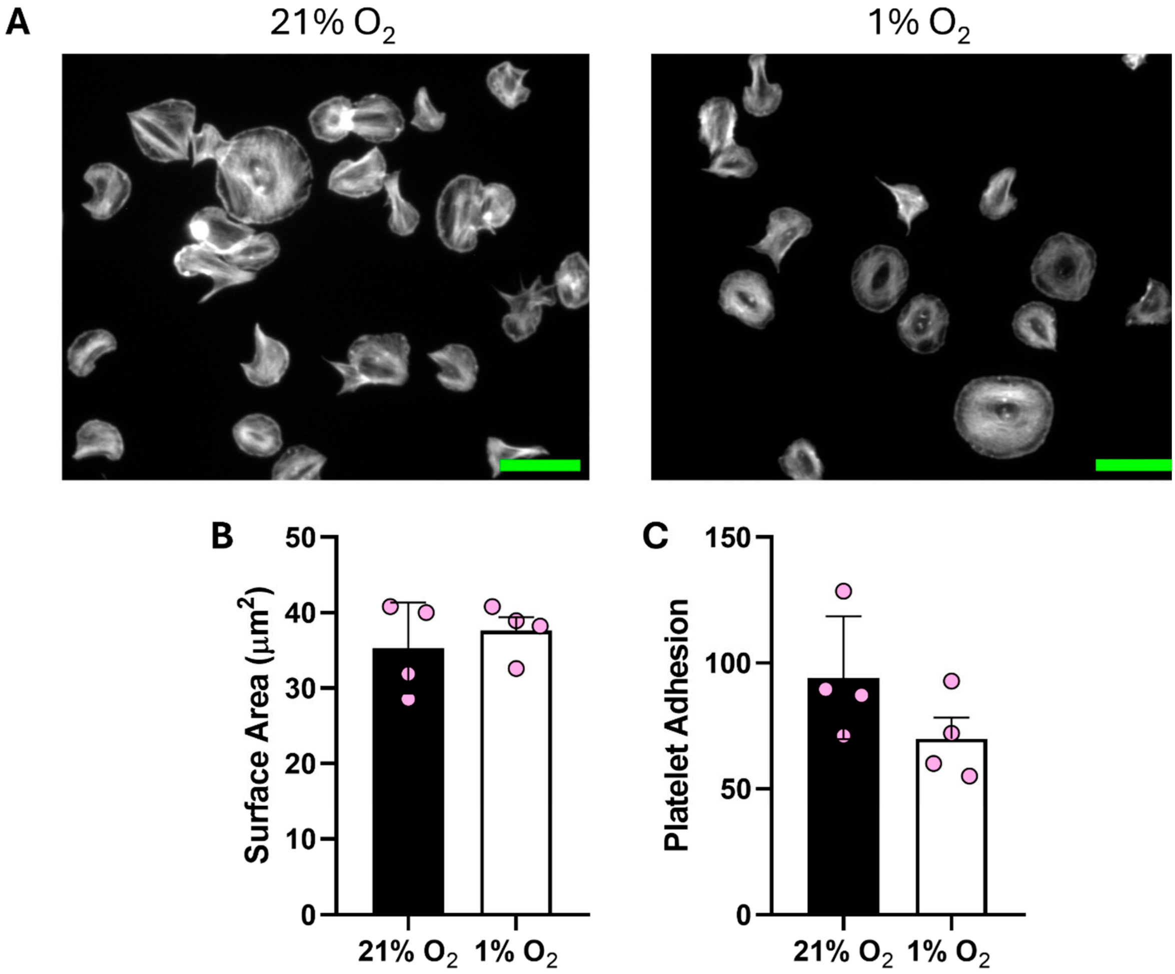

Figure S2: Platelet spreading on CRP-A is unaffected by hypoxic conditions. *Washed platelets (2x10*^{7} *platelets/mL) were spread on a CRP-A matrix (100 µg/ml) for 25 minutes before being fixed with PFA,*

membrane permeabilization with Triton X-100 and staining with FITC-phalloidin. A) Representative images of platelet spreading on CRP-A at 21%, and 1% oxygen with scale bar showing 10 µm. Graphs showing B) platelet surface area and C) average platelet adhesion. Data are presented as mean ± SEM. n= 4. Statistical analysis was calculated using a paired t-test.

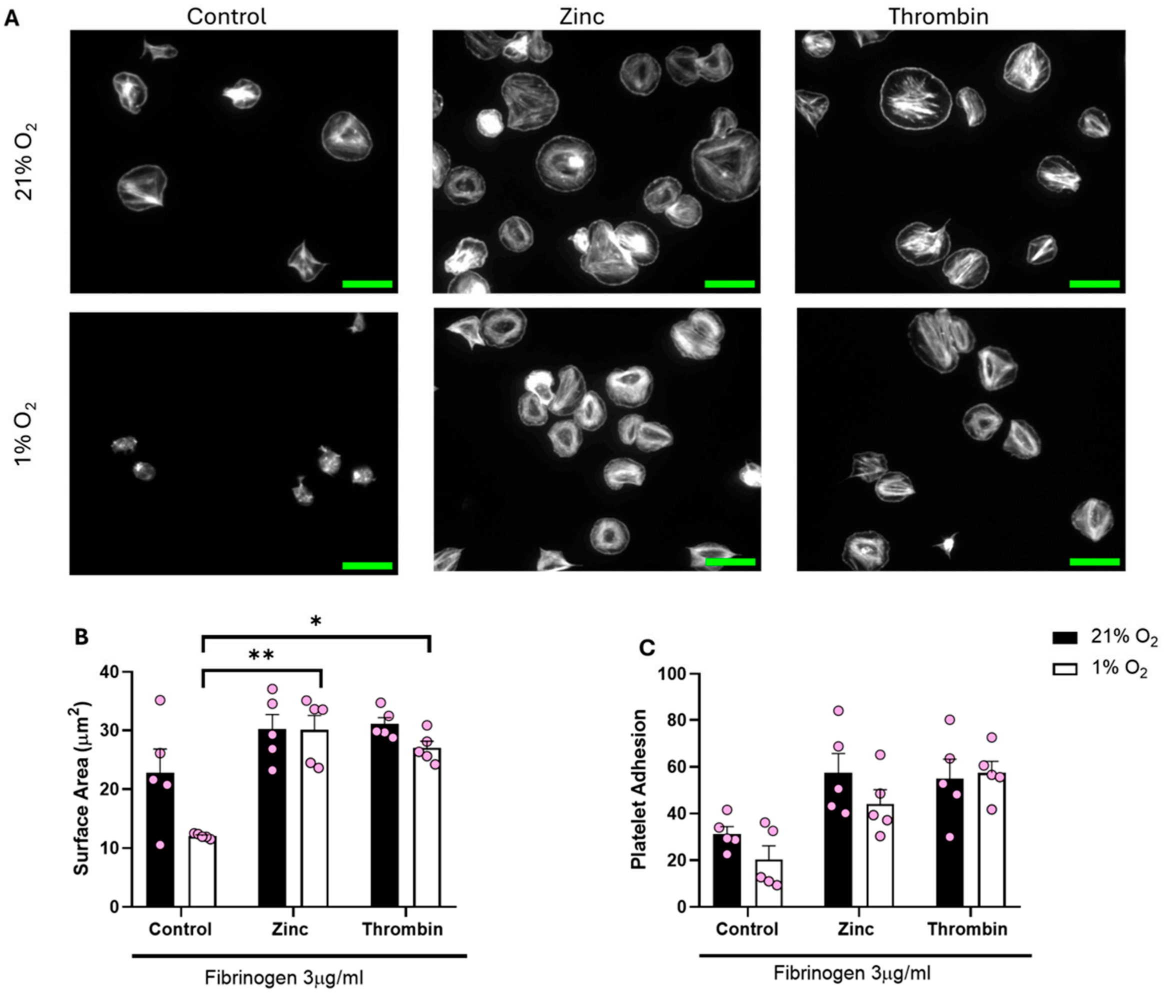

Figure S3: Platelet spreading on fibrinogen (3 µg/ml) at 1% O_2 levels is restored following pretreatment with zinc and thrombin. *Washed platelets (2x10^7 platelets/ml) were preincubated with zinc (100 µM) and thrombin (0.1 U/ml) for 2 minutes before spreading on fibrinogen (3 µg/ml) for 25 minutes before being fixed with PFA, membrane permeabilised with Triton X-100, and stained with FITC-phalloidin. (A) Representative images of platelets spread on fibrinogen at 21% and 1% O_2, with scale bar showing 10 µm. Graphs show (B) average platelet surface area and (C) average number of platelets adhered. Data are presented as mean ± SEM. n= 5. Statistical analysis was calculated using a repeated measures two-way ANOVA with Tukey's post hoc multiple comparisons test. *p<0.05, **p<0.01.*

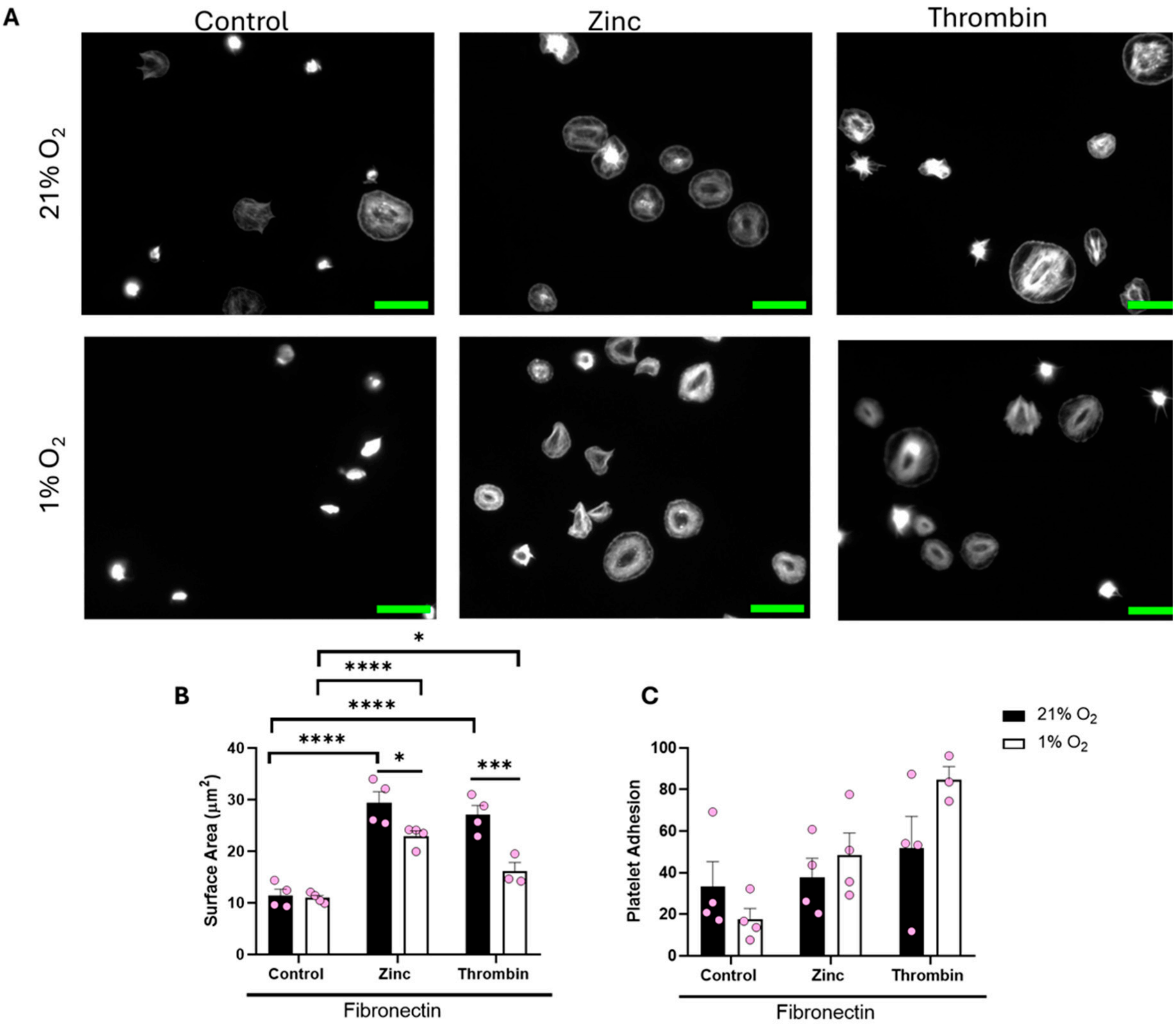

Figure S4: Platelet spreading on fibronectin is increased when platelets are treated with zinc or thrombin. *Washed platelets ($2x10^7$ platelets/ml) were spread on fibronectin (100 µg/ml) for 25 minutes at 1% and 21% O_2 before being fixed with PFA, permeabilized with Triton X-100, and stained with FITC-phalloidin. (A) Representative images of platelets spread on fibronectin at 21% and 1% O_2, with scale bar showing 10 µm. Graphs show (B) average platelet surface area and (C) average number of platelets adhered. Data are presented as mean ± SEM. n= 4. Statistical analysis was calculated using a repeated measure two-way ANOVA with Tukey's post hoc multiple comparisons test. *p<0.05, ***p<0.001, ****p<0.0001*

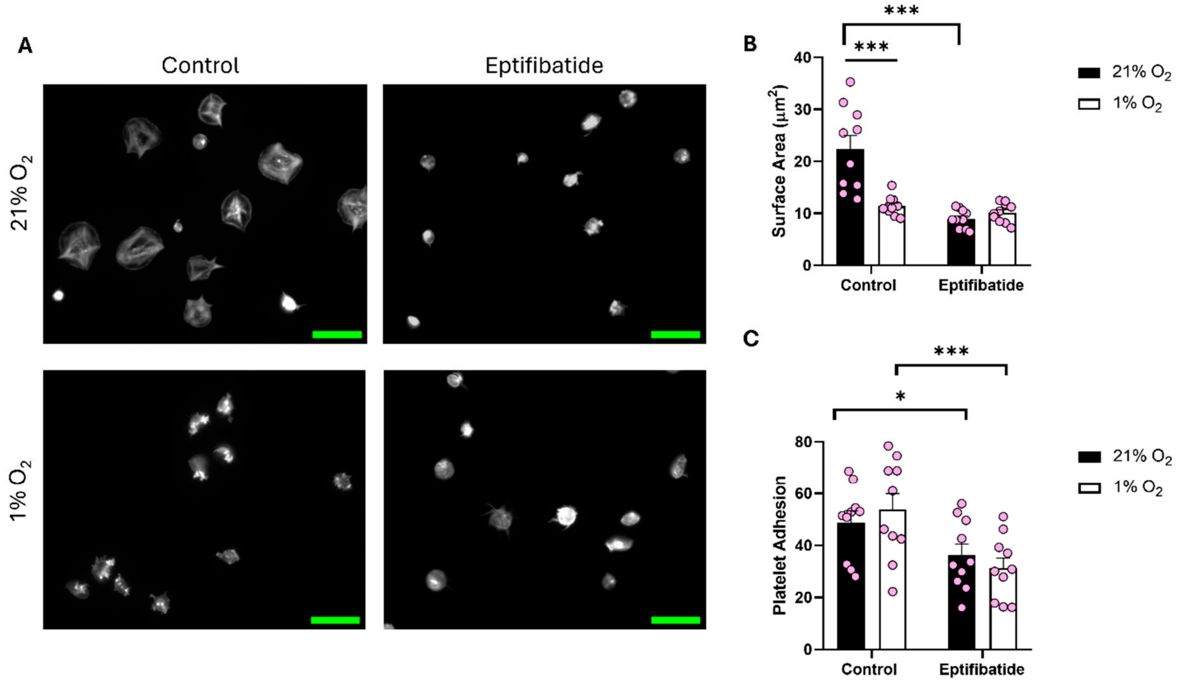

Figure S5: Platelet spreading following eptifibatide treatment is unaffected at 1% O_2. *Washed platelets ($2x10^7$ platelets/ml) were treated with eptifibatide (9µM) before being spread on fibrinogen* (100 µg/ml) *for 25 minutes, fixed with PFA, membrane permeabilised with Triton X-100 and stained with FITC-phalloidin. A) Representative images of platelet spreading on fibrinogen at 21% and 1% O_2 in the presence of eptifibatide with scale bar showing 10 µM B) graphs showing platelet surface area and C) platelet adhesion. Data presented as mean +/- SEM. n=10. Statistical analysis calculated using a repeated measures two way ANOVA, *p<0.05, ***p<0.001.*

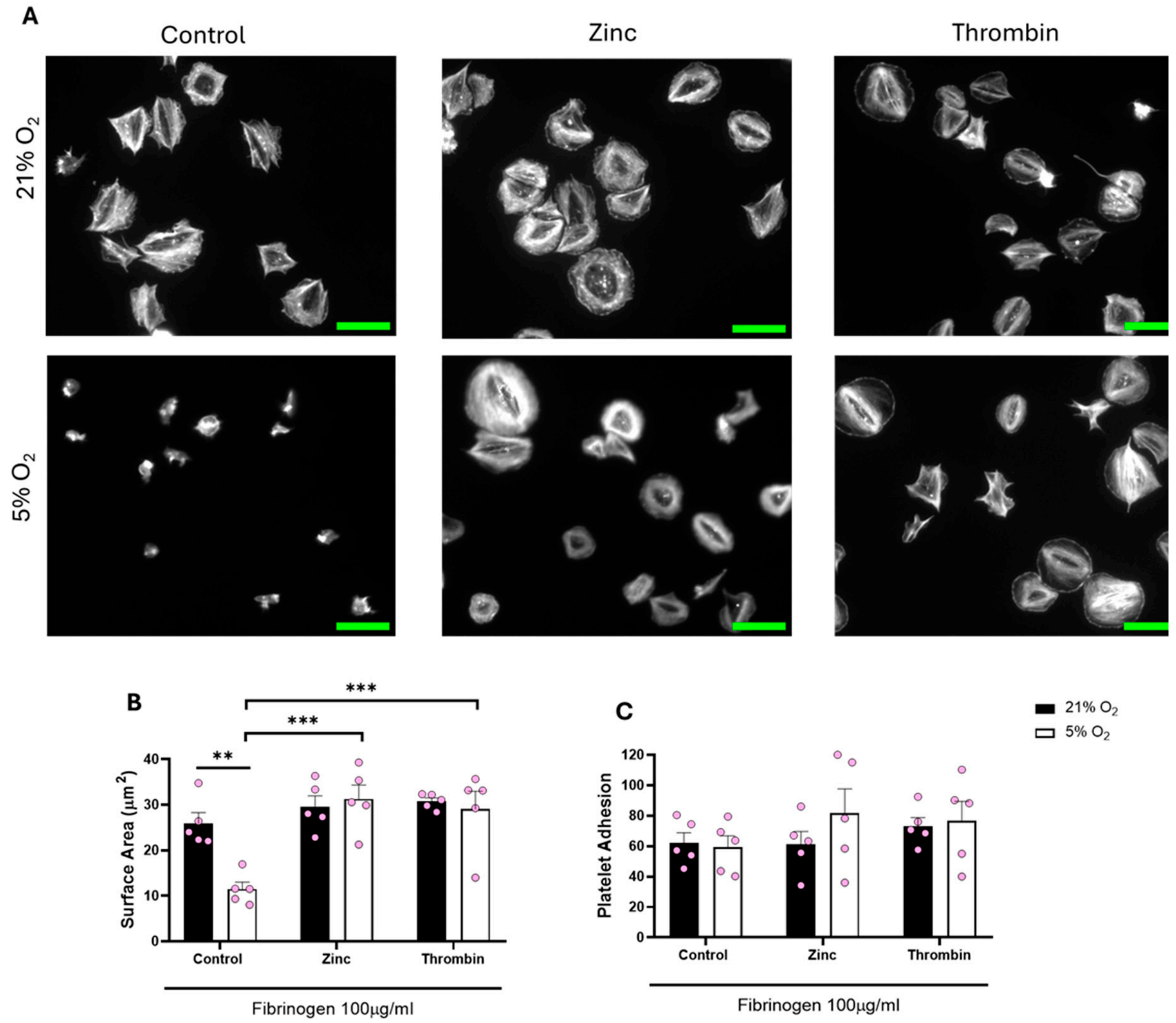

Figure S6: Platelet spreading on high density fibrinogen at venous O_2 levels is restored following pretreatment with zinc and thrombin. *Washed platelets ($2x10^7$ platelets/ml) were preincubated with zinc (100 µM) and thrombin (0.1 U/ml) for 2 minutes before spreading on fibrinogen (100 µg/ml) for 25 minutes before being fixed with PFA, permeabilised with Triton X-100, and stained with FITC-phalloidin. (A) Representative images of platelets treated with zinc, thrombin and control at 21% and 5% O_2, with scale bar showing 10 µm. Graphs showing (B) average platelet surface area and (C) platelet adhesion. Data are presented as mean ± SEM. n= 5 Statistical analysis was calculated using repeated measures two-way ANOVA with a Tukey's post hoc test. **p<0.01, ***p<0.001*

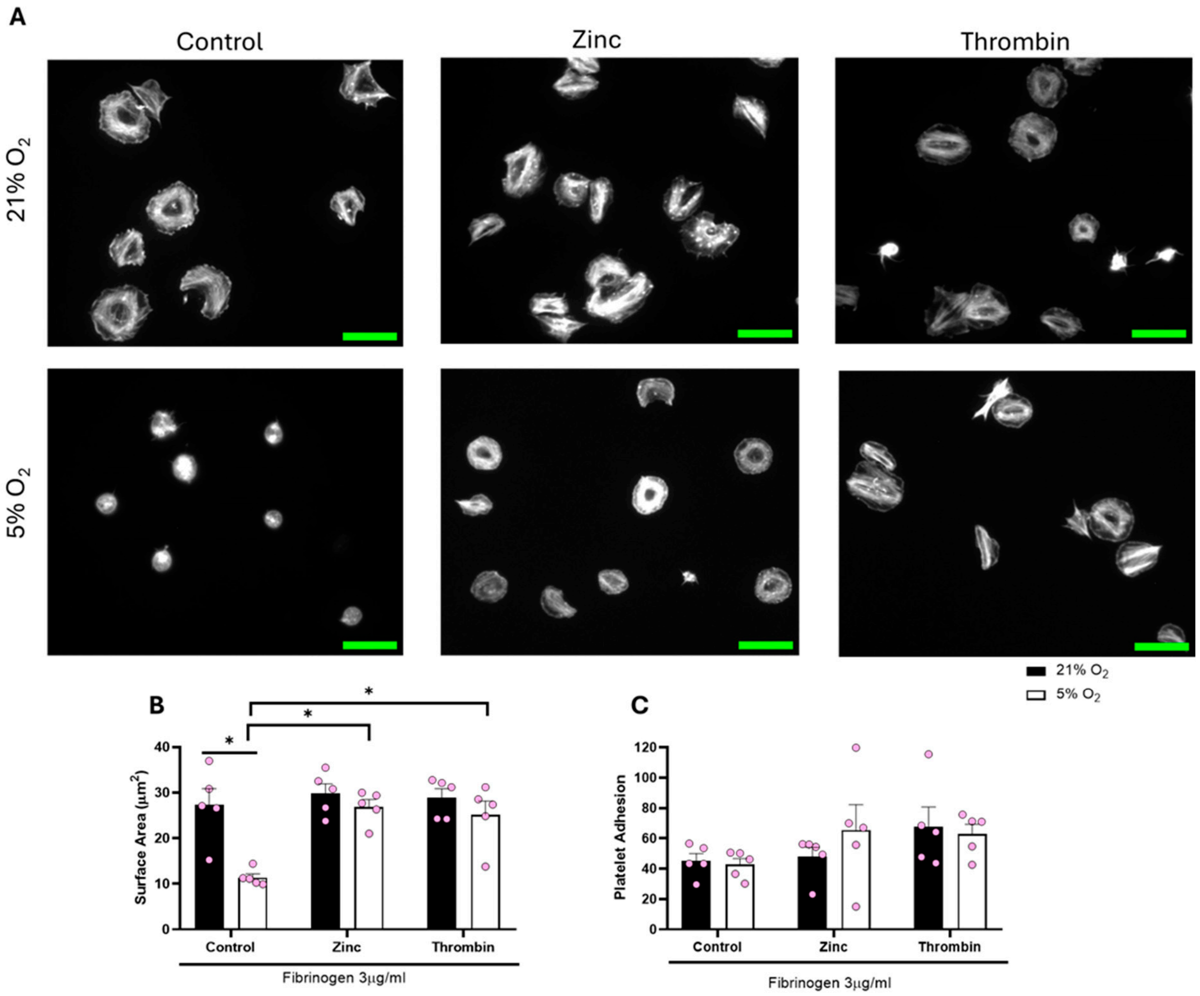

Figure S7: Platelet spreading on low density fibrinogen at venous O_2 levels is restored following pretreatment with zinc and thrombin. *Washed platelets (2x10^7 platelets/ml) were preincubated with zinc (100 µM) and thrombin (0.1 U/ml) for 2 minutes before spreading on fibrinogen (3 µg/ml) for 25 minutes before being fixed with PFA, permeabilised with Triton X-100, and stained with FITC-phalloidin. (A) Representative images of platelets treated with zinc, thrombin and control at 21% and 5% O_2, with scale bar showing 10 µm. Graphs showing (B) average platelet surface area and (C) platelet adhesion. Data are presented as mean ± SEM. n= 5 Statistical analysis was calculated using repeated measures two-way ANOVA with a Tukey's post hoc test. *p<0.05.*

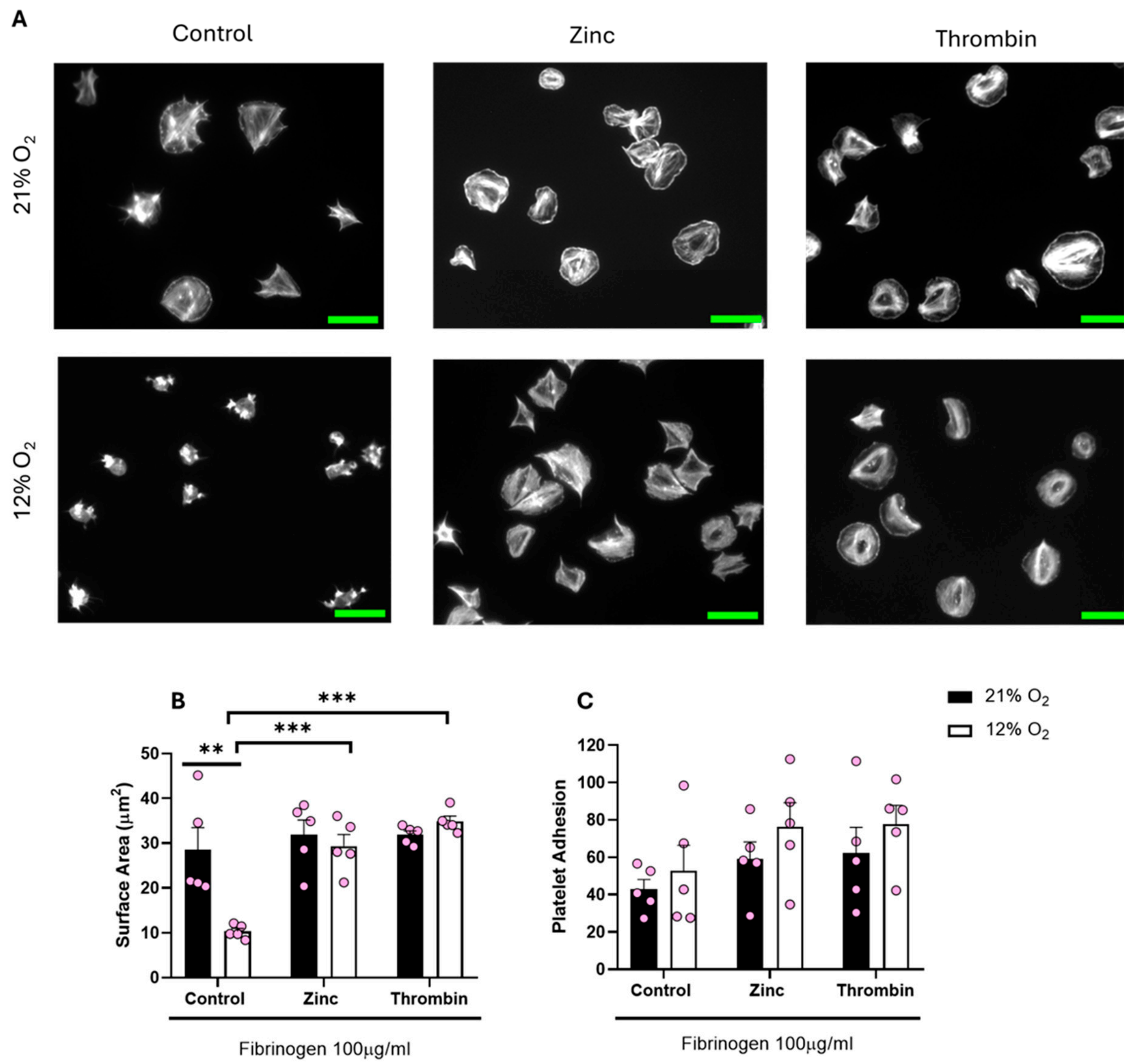

Figure S8: Platelet spreading on high density fibrinogen at arterial O_2 levels is restored following pretreatment with zinc and thrombin. *Washed platelets (2x10^7 platelets/ml) were preincubated with zinc (100 µM) and thrombin (0.1 U/ml) for 2 minutes before spreading on fibrinogen (100 µg/ml) for 25 minutes before being fixed with PFA, permeabilised with Triton X-100, and stained with FITC-phalloidin. (A) Representative images of platelets treated with zinc, thrombin and control at 21% and 12% O_2, with scale bar showing 10 µm. Graphs showing (B) average platelet surface area and (C) platelet adhesion (C). Data are presented as mean ± SEM. n= 5 Statistical analysis was calculated using repeated measures two-way ANOVA with a Tukey's post hoc test. **p<0.01, ***p<0.001.*

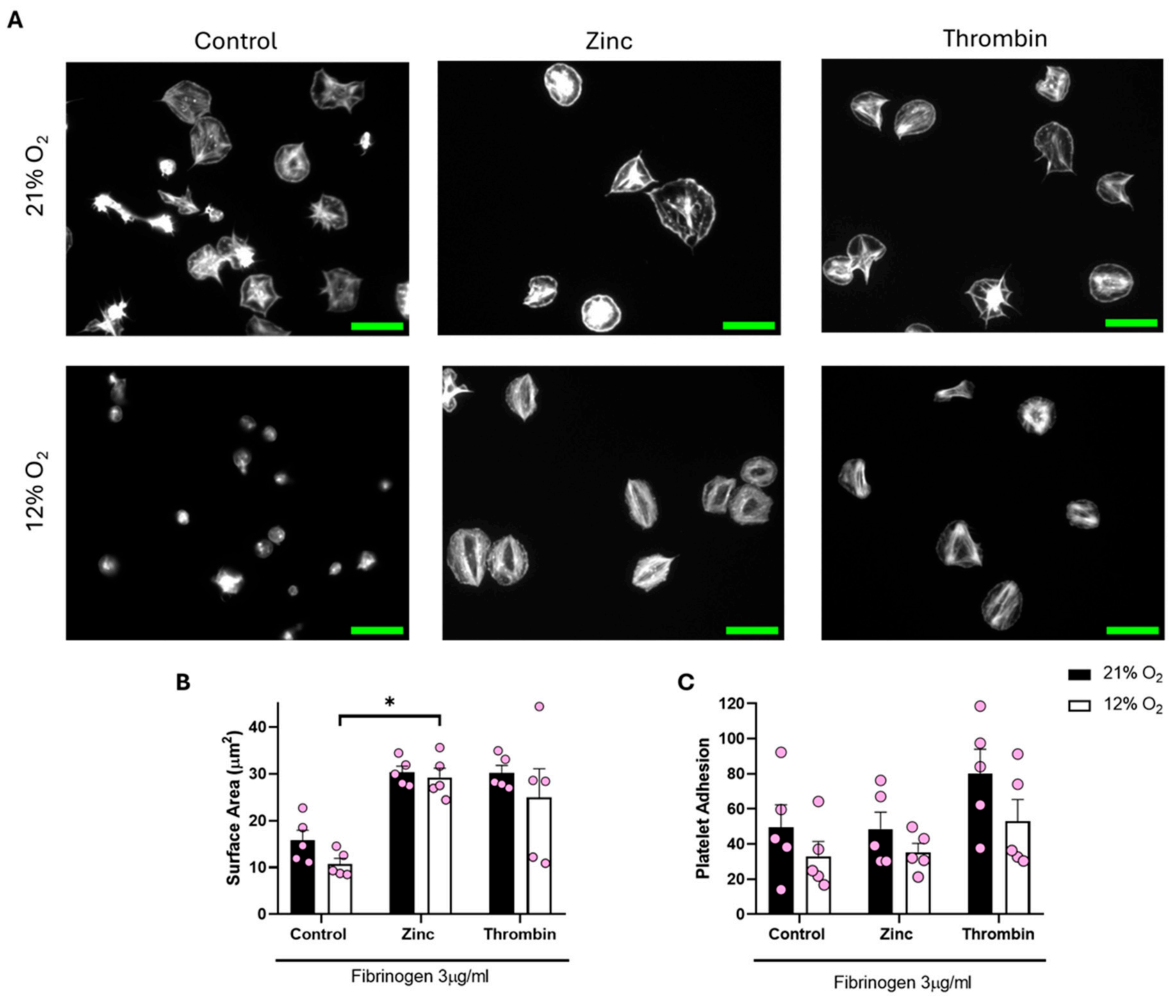

Figure S9: Platelet spreading on low density fibrinogen at arterial O_2 levels is restored following pretreatment with zinc and thrombin. *Washed platelets ($2x10^7$ platelets/ml) were preincubated with zinc (100 µM) and thrombin (0.1 U/ml) for 2 minutes before spreading on fibrinogen (3 µg/ml) for 25 minutes before being fixed with PFA, permeabilised with Triton X-100, and stained with FITC-phalloidin. (A) Representative images of platelets treated with zinc, thrombin and control at 21% and 12% O_2, with scale bar showing 10 µm. Graphs showing (B) average platelet surface area and (C) platelet adhesion. Data are presented as mean ± SEM. n= 5 Statistical analysis was calculated using repeated measures two-way ANOVA with a Tukey's post hoc test. *p<0.05.*

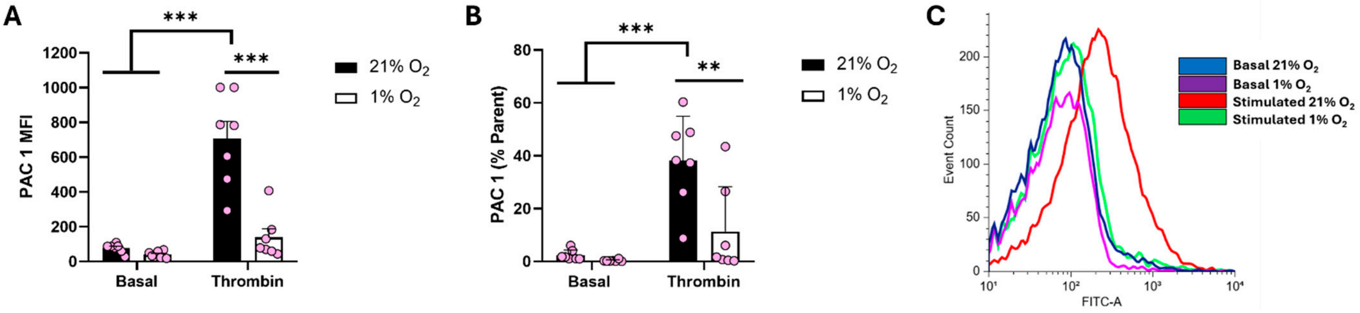

Figure S10: Integrin $\alpha_{IIb}\beta_3$ function is markedly reduced in hypoxic platelets. *Washed platelets (1x10^7 platelets/ml) were stained with monoclonal antibodies for CD42b and PAC1 and treated with thrombin (0.1 U/ml) for 20 minutes. (A) Mean fluorescent intensity (MFI), (B) % Parent for PAC-1 and (C) representative histograms of PAC1 expression. n=7. Data shown as mean Data shown as mean ± SEM (A) and SD (B). Statistical significance was calculated using a two-way ANOVA followed by a Tukey's post-hoc test. **p <0.01 ***p<0.001.*

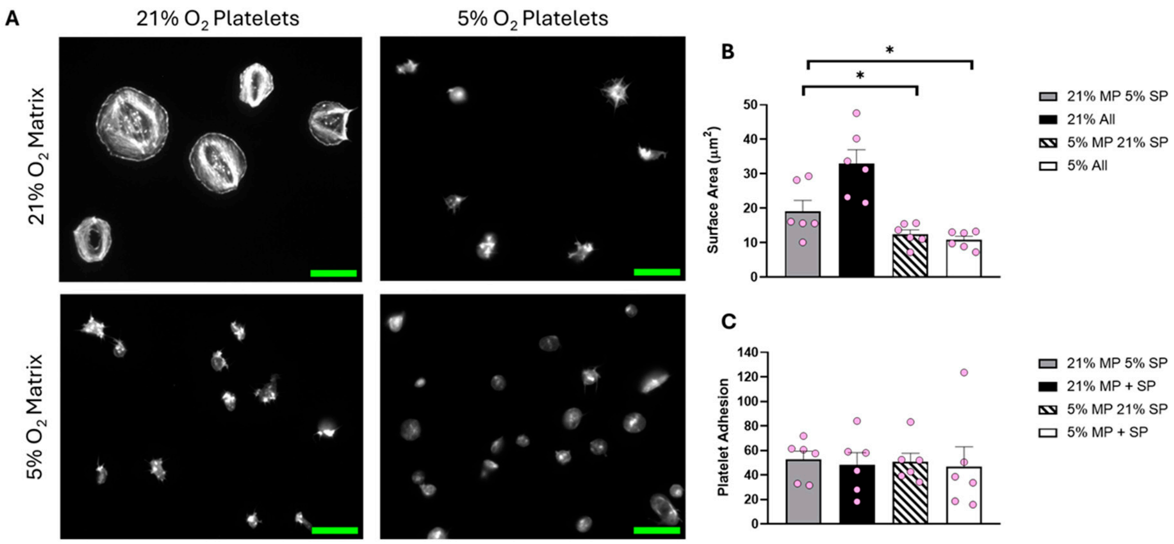

Figure S11: Location of fibrinogen matrix preparation plays an important factor in platelet spreading. *Fibrinogen (100 µg/ml) matrices were prepared at both 21% and 5% O_2, platelet spreading was then conducted using washed platelets kept at 21% and 5% O_2. Washed platelets (2x10^7 platelets/ml) were spread on fibrinogen for 25 minutes before fixation with PFA, permeabilization using triton x-100, and staining with FITC-phalloidin. (A) Representative images of platelet spreading at each condition, with scale bar showing 10 µm. Graphs show (B) average platelet surface area and (C) platelet adhesion. Data are presented as mean ± SEM. n= 6. Statistical analysis was calculated using repeated measures one-way ANOVA. *p<0.05. Matix prep (MP), spread platelets (SP).*

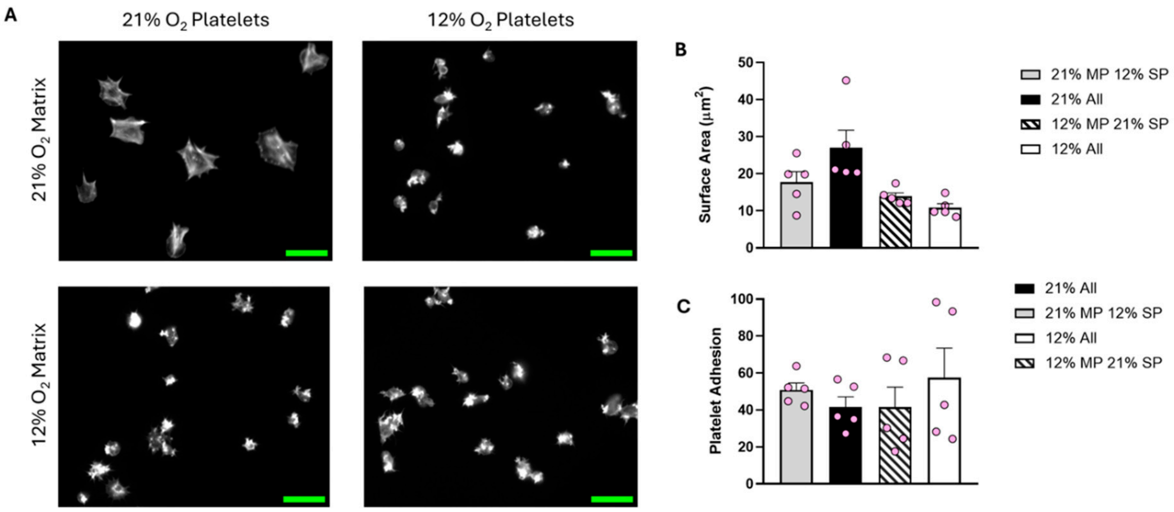

Figure S12: Location of fibrinogen matrix preparation plays an important factor in platelet spreading. *Fibrinogen (100 µg/ml) matrices were prepared at both 21% and 12% O_2, platelet spreading was then conducted using washed platelets kept at 21% and 12% O_2. Washed platelets ($2x10^7$ platelets/ml) were spread on fibrinogen for 25 minutes before being fixated with PFA, permeabilized using Triton X-100, and stained with FITC-phalloidin. (A) Representative images of platelet spreading at each condition, with scale bar showing 10 µm. Graphs show (B) average platelet surface area and (C) platelet adhesion. Data are presented as mean ± SEM. n= 5. Statistical analysis was calculated using repeated measures one-way ANOVA. Matix prep (MP), spread platelets (SP).*

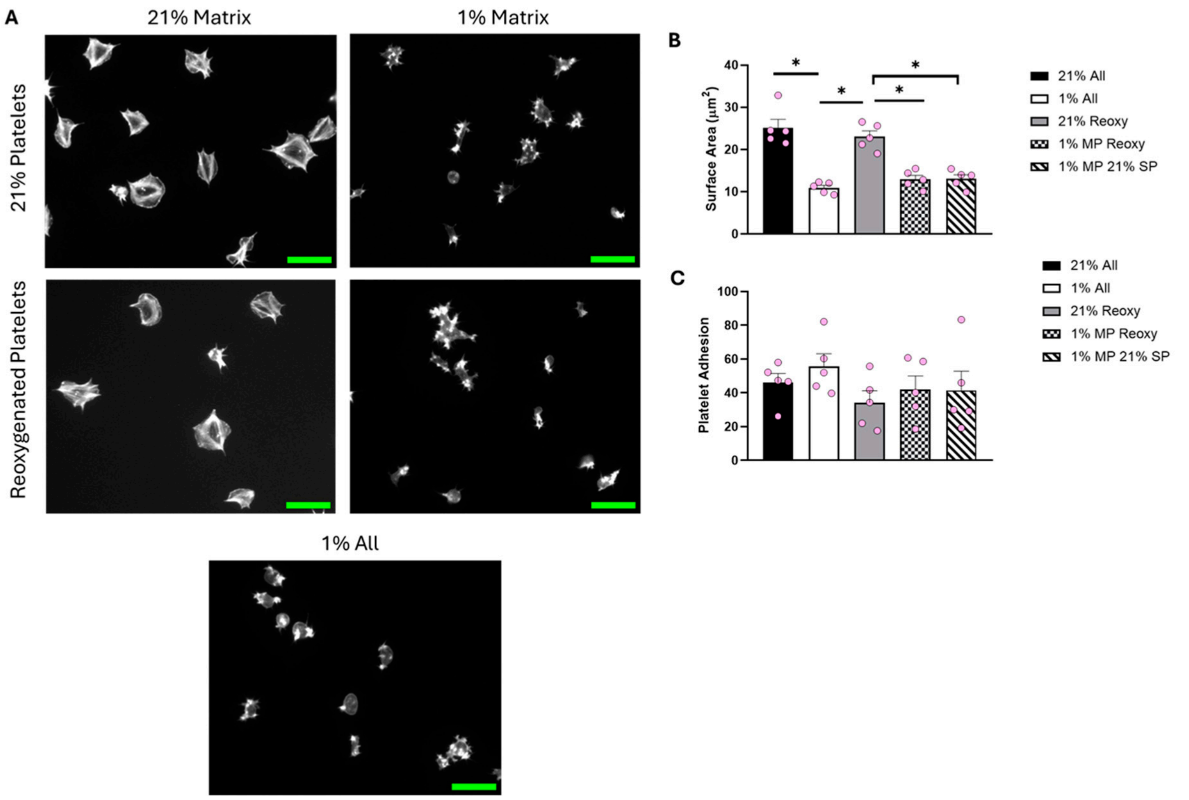

Figure S13: Platelet spreading on fibrinogen is restored following reoxygenation of platelets. *Fibrinogen (100 µg/ml) matrices were prepared at both 21% and 1% O_2, platelet spreading was conducted using washed platelets kept at 21%, 1% or reoxygenated platelets following a 2 hour incubation period at 1% O_2. Washed platelets ($2x10^7$ platelets/ml) were spread on fibrinogen for 25*

*minutes before being fixed with PFA, membrane permeabilised using Triton X-100, and then stained with FITC-phalloidin. (A) Representative images of platelet spreading at each condition, with scale bar showing 10 µm. Graphs show (B) average platelet surface area and (C) platelet adhesion. Data presented as mean ± SEM. n= 5. Statistical analysis was calculated using repeated measures one-way ANOVA *p<0.05. Matix prep (MP), spread platelets (SP), reoxygenated platelets (reoxy).*

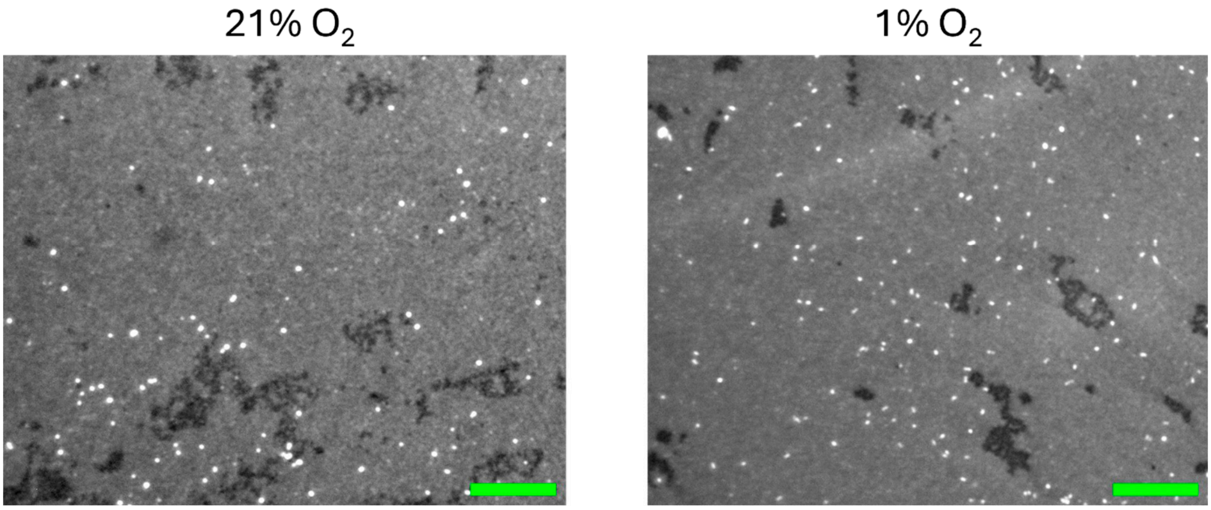

Figure S14: Formation of a fibrinogen monolayer is unaffected by oxygen concentration. *Alexa Fluor 647 labelled fibrinogen (100 µg/ml) was coated onto glass coverslips at 21% and 1% O_2 for 1 bour, followed by blocking with BSA (5 mg/ml) and fixation with PFA (4%). Representative images of the fibrinogen monolayer were captured on a Zeiss Axio Image fluorescence microscope with a x63 oil immersion objective (1.4 NA) using Zen Pro software (Zeiss). n=3.*
